# Brain extraction for fixed tissue banking: a technical report

**DOI:** 10.17879/freeneuropathology-2026-9411

**Published:** 2026-05-26

**Authors:** Mads Wolf, Autumn Beck, Laura Paredes, Sarah Darcy, Alexander Parra, Gabriel A. Taylor, Macy Garrood, Emma L. Thorn, Claudia De Sanctis, John F. Crary, Kurt Farrell, Andrew T. McKenzie

**Affiliations:** 1 Apex Neuroscience, Salem, Oregon, USA; 2 Friedman Brain Institute, Departments of Pathology, Neuroscience, and Artificial Intelligence & Human Health, Icahn School of Medicine at Mount Sinai, New York, New York, USA; 3 Neuropathology Brain Bank & Research Core and Ronald M. Loeb Center for Alzheimer's Disease, Icahn School of Medicine at Mount Sinai, New York, New York, USA

**Keywords:** Brain extraction, Tissue preservation, Brain banking, Immersion fixation, Postmortem changes

## Abstract

Brain banking enables the study of human neural tissue and is essential for research on disease, physiology, and neuroanatomy. An essential step for whole brain studies is the extraction of the brain from the skull. Yet detailed technical descriptions of brain removal are rare, perhaps contributing to the artifactual tissue disruption sometimes observed as a result of the procedure. Here, we describe a method for whole brain extraction that could be implemented in the context of a whole-body donation, focused on the use case of fixed tissue banking. The method involves a sequential craniectomy that uses both circumferential and midline sagittal cuts, followed by a posterior approach to cutting the dura. We report the application of this protocol across n = 105 human whole-body donors. We document the time required for each procedural step and the frequency of craniectomy artifacts and skull edge artifacts at the brain surface. When the brain tissue is particularly soft, we also describe the potential use of *in situ* immersion fixation to increase tissue stiffness before removal, finding however that this also slows the diffusion of chemicals into the interior of the brain. Our experience suggests that the effectiveness of brain extraction can be improved via procedural optimizations. Technicians can become comfortable with the method after approximately 5–10 cases. We anticipate that improving brain extraction quality may support downstream work in the development of diagnostics and treatments for neurological and psychiatric disease.

## Abbreviations

**CT** - Computed Tomography, **H&E** - Hematoxylin and Eosin, **IRB** - Institutional Review Board, **PMI** - Postmortem Interval, **SD** - Standard deviation, **WSI** - Whole slide image.

## Introduction

Brain banking plays a critical role in advancing our understanding of the human brain in both health and disease. The systematic collection and preservation of whole brains enables researchers to examine neuroanatomical structures, pathological changes, and molecular alterations that underlie neurological and psychiatric conditions (1). Prior to study, the brain must be extracted from the skull. This is necessary to facilitate detailed gross examination, dissection of structures across the brain, and long-term banking. Various methods have been developed over the years to accomplish this, each with advantages and disadvantages depending on the specific clinical, research, or educational goals. For example, while hand saws were commonly used historically for the craniectomy portion of brain banking, the development of electric autopsy saws enabled the procedure to be performed more rapidly (5). The brain isolation procedure can also be performed with different cuts along different parts of the skull, which have different relative costs and benefits in terms of time and the likelihood of causing damage to different parts of the brain during extraction (6).

There are multiple factors to consider when optimizing brain extraction procedures for research applications. Perhaps most critical is the trade-off between speed of extraction and tissue artifacts. The postmortem interval (PMI) between death and tissue preservation inevitably leads to progressive autolytic changes that can be appreciated at the ultrastructural level (12). As a result, rapid autopsy programs aim to minimize PMI to preserve molecular integrity. It has been reported that autopsy tissue can approach the quality levels of surgical biopsy specimens when samples are processed rapidly, although this undoubtedly depends on the aspect of the tissue being studied (13). However, faster speed of extraction must be weighed against the risk of causing more mechanical damage during removal. Rushing the extraction process can result in tissue artifacts, including transverse saw or osteotome artifacts in the parenchyma, artifacts due to the contact of brain tissue with the skull edges, mechanical stress on the brain parenchyma due to pulling motion, and damage to structures at the skull base such as the cranial nerves (9,11,14).

Another important factor to consider is the biological state of the brain being removed. The stiffness of the brain at the time of extraction – which is influenced by the PMI, agonal factors, myelin content, and to what extent the tissue has been perfusion-fixed – affects how readily the brain can be removed without artifacts (15,16). Softer brains, whether due to longer PMI, decomposition, or reduced myelination in certain populations, are more susceptible to deformation and damage during manipulation (16).

Different brain banks have different procedural constraints and goals. In our case, we generally perform our brain extraction procedure on the isolated cephalon. We use the cephalon preparation to perfuse the brain through the carotid and/or vertebral arteries, with the goal of preserving its fine structure for downstream applications (17). Notably, isolation of the cephalon is a technique that has long been used to aid in the study of the brain, dating back to early anatomists like Mondino de Liuzzi and Andreas Vesalius (18,19), and remains useful in modern contexts such as neurosurgical education (20). This approach enables easier re-positioning of the specimen throughout extraction, potentially reducing tissue artifacts and thereby improving the utility of the brain specimen for research purposes. However, we note that the vast majority of brain extraction protocols do not involve prior cephalon isolation (23). This is in part because most brain banks receive brain-only donations, among other factors. Still, several aspects of our protocol may translate to standard brain extraction procedures.

In minke whales, which have large brains, one approach has been to perform the initial immersion fixation of the brain *in situ*, through an opening in the skull, prior to brain removal (24). A similar protocol on manatee calves combined *in situ* injection of fixative through the foramen magnum and immersion fixation prior to brain extraction (25). *In situ* immersion fixation prior to removal has also been reported in rodent experiments (26). Additionally, when brains are first perfusion fixed, leaving them *in situ* for a period of time prior to extraction has been described as a way to decrease damage attributed to extraction (14,27,28). This method may be especially useful to mitigate damage during the extraction of soft brains. However, to the best of our knowledge, the potential utility of immersion fixation *in situ* without prior perfusion in human brain banking has not yet been investigated.

In this technical report, we describe our approach to brain extraction. We detail the procedural steps, report our timing data and tissue artifact rates, and discuss the learning curve for technicians new to the method. We also present preliminary findings on *in situ* immersion fixation as a strategy for stiffening soft brains prior to removal, including a CT-based assessment of its effects on chemical diffusion. By sharing these methods and observations, we hope to contribute to the ongoing refinement of brain banking practices, with the aim of improving the quality of tissue collection for neuroscience research.

## Methods

### Anatomical donation procedures

Anatomical whole-body donations were coordinated by a partner whole body donation organization operating as a Nontransplant Anatomical Research Recovery Organization under Oregon Health Authority regulations. All donations were processed following informed consent procedures. The Apex Neuroscience Brain and Tissue Bank operates under an exemption determination issued by the Pearl Institutional Review Board (IRB) after submission of our protocols for review (Pearl IRB ID #2023-0260).

### Specimens and personnel

A total of 105 brain extractions were included in the analysis. Procedures were performed by 8 technicians, with case volumes ranging from 2 to 33 extractions per technician. All technicians held at least an associate’s or bachelor’s degree, generally in the biomedical sciences. One technician (Tech 4) had substantial prior brain extraction experience before this case series. The remaining 7 technicians had no significant prior brain extraction experience and were trained during this study.

### Initial dissection

**Figure 1:**
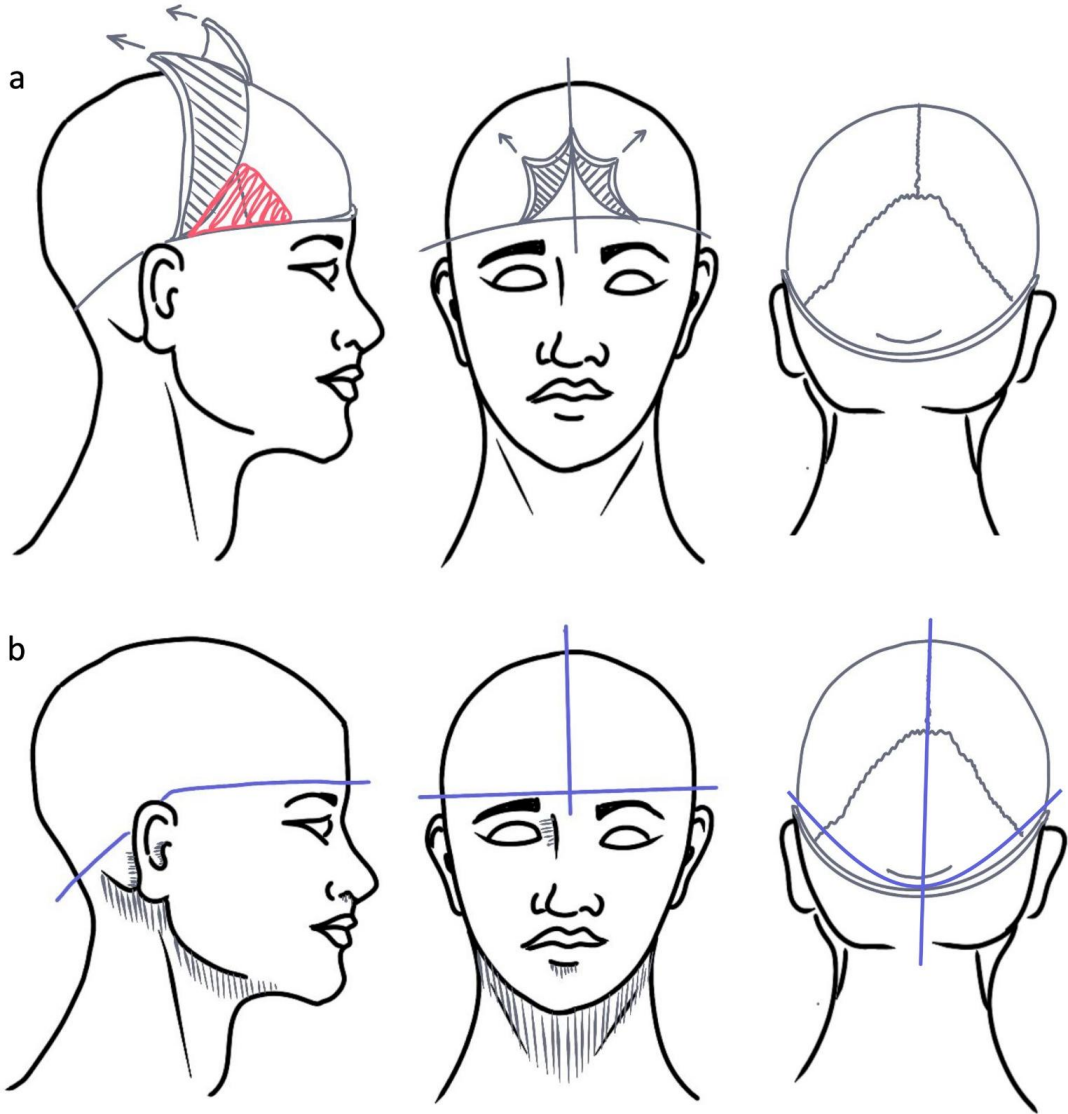
**a**: Diagram of the soft tissue dissection procedure. **b**: Diagram of the craniectomy procedure. The craniectomy is done following similar lines as the soft tissue removal, aiming for approximately the blue lines in this diagram.

The cephalon is isolated from the body via dissection at the level of the laryngeal prominence. When cephalon isolation is not performed, the head can be elevated on a standard autopsy head block with the body supine, and the same removal steps can be applied in that position. In most cases, we perform perfusion of the cephalon through the internal carotid arteries (and possibly also the vertebral arteries), as previously described (17). After perfusion is completed (or if it is not performed), we then begin the brain extraction process with a circumferential scalp incision along the brow line with a scalpel (**Figure 1a**; **Table 1**). This extends posteriorly behind the ears to the base of the skull beneath the occipital protuberance. We then make an intersecting incision through the scalp across the sagittal line, forming an inverted "T" shape between the eyebrows. Note that we use the brow line incision because our donors are not reconstructed for viewing. When cosmetic preservation is required, a coronal incision from ear to ear posterior to the hairline is a commonly used alternative, with the scalp reflected anteriorly and posteriorly to expose the cranium.

The pieces of scalp are then removed from the exterior of the cranium and any remaining soft tissue, such as any exposed frontalis, temporalis, or occipitalis muscle, is removed to increase access to the bone and reduce snag points for future saw cuts. Once the excess tissue is removed, we then score the intended saw-cut lines (both circumferential and sagittal) with a scalpel to assist in keeping a steady, even line cut.

**Table 1:** Supplies used for brain extraction

Equipment	Supplier	Product identifier
Scalpel Handle No 3	MedHelp	B08QDMFJRG
Feather Scalpel Blade #22	Electron Microscopy Sciences	72044-22
Mopec 810 Autopsy Saw	Mopec	BD810
Standard Large Section Pathology Saw Blade	Mopec	BD101-P
Skull Breaker, 3"	Integra Miltex	34-210
Lexer Bone Osteotome 15 mm x 22 cm with Fiber Handle	Medikrebs	TT283
Heavy Bone Mallet	Surgical Online	B07CRQTXDW
Brain Spatula, Malleable, 16 mm x 20 cm	Medikrebs	B07QW3MZH6
Ferris Smith Tissue Forceps, 7", Serrated Platform Tips with 2 x 3 Teeth	Integra Miltex	26-958
Standard Surgical Scissors, Blunt/Blunt, Curved	Fine Science Tools	14003-13
Mayo Dissecting Scissors, Extra Long, Curved, 41 cm	Carnegie Surgical	510-100

### Craniectomy technique

Following the scored lines, we use an oscillating saw to begin cutting through the cranium using a "gliding" method (**Figure 1b**). This method employs a lighter back-and-forth motion through the bone to prevent the saw from cutting completely through the bone and lacerating the dura and cortical tissue. Skull thickness varies across different regions of the skull, being thicker in parts of the frontal and occipital regions and thinner in parts of the temporal region, which increases the risk of accidentally plunging through the bone and causing a craniectomy artifact. The goal is not to cut completely through the bone at once, but rather to create a guiding line for the osteotome to complete the calvaria separation. As with the soft tissue removal, the skull is cut at approximately 3–4 cm below the occipital protuberance, just above where the soft tissue is removed. Note that we do not make an additional "hinge" cut in the occipital region (10), but merely make the circumferential cut more inferior than in most other described protocols. After the saw cut is completed, we then switch to an osteotome and mallet to separate the final bone bridges, using a light hand and lever action to ensure the osteotome does not plunge through the bone and into the brain tissue. The characteristic auditory feedback, often likened to the sound of a coconut cracking open by our technicians, allows us to ensure full separation along the guideline. We then carefully remove the two pieces of bone to expose the dura, separating any stubborn attachments with a malleable spatula by scraping along the interior of the calvaria or cutting with curved blunt-tip dissection scissors.

### Dura removal

**Figure 2:**
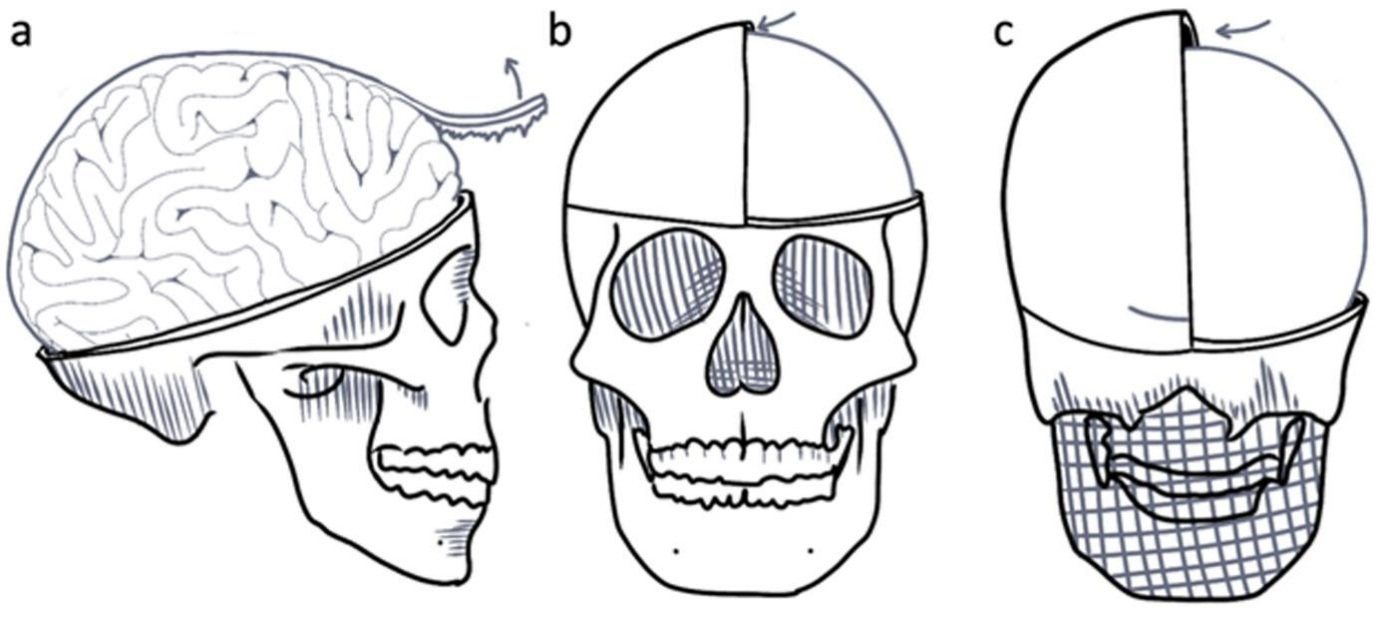
Diagram of steps related to the dura removal procedure. **a**: The removal of the falx cerebri with Adson forceps and scissors. **b**, **c**: Arrows indicate the direction that the malleable spatula is inserted to free the dural attachments from the interior of the skull, when necessary.

**Figure 3:**
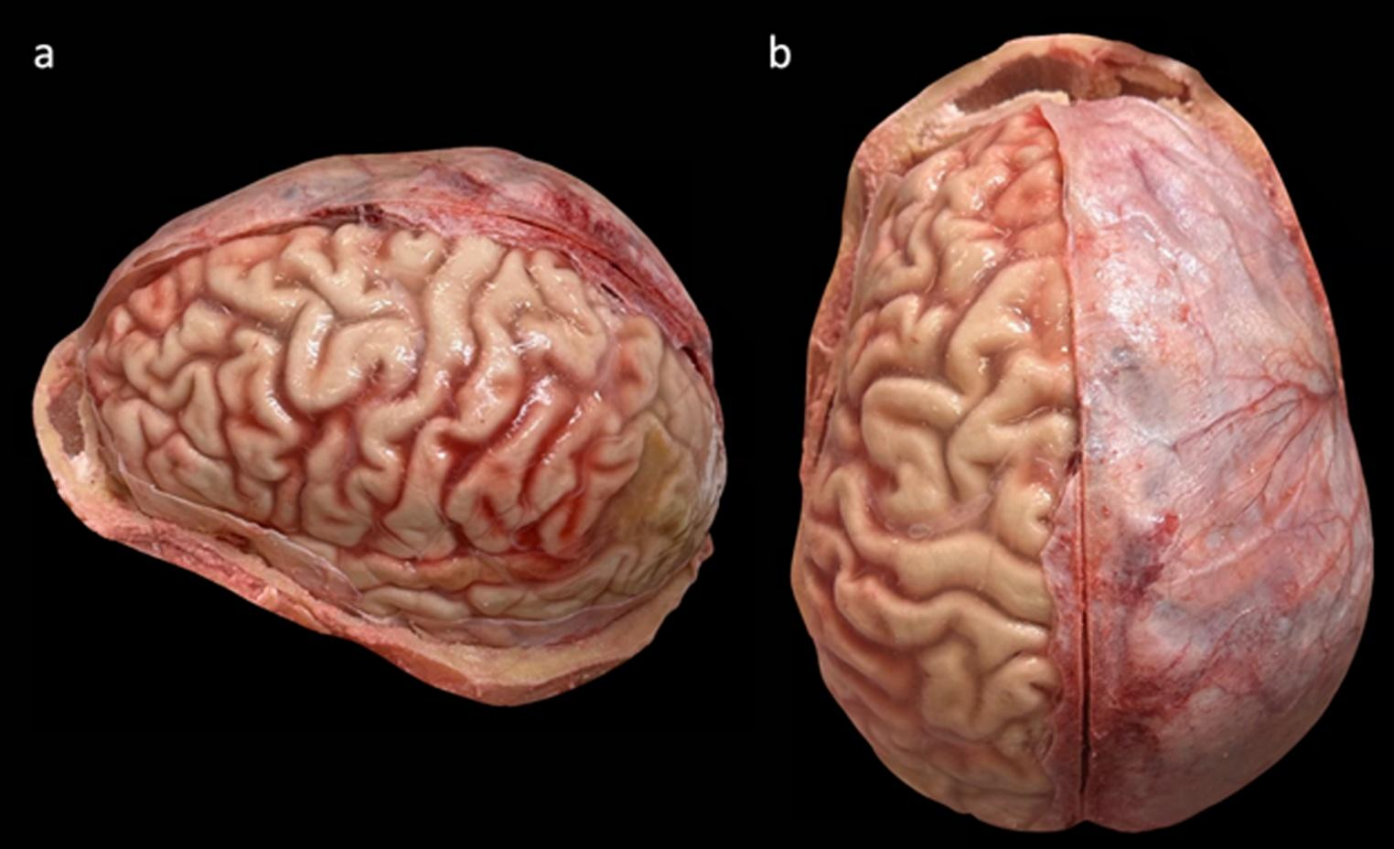
Example images showing a brain part of the way through the dural removal process. **a**: Lateral view showing the left hemisphere with the dura removed. **b**: Superior view showing the dura remaining on the right hemisphere. Donor #325, a 35-year-old female donor with a PMI of 35 hours.

If the previous step was done correctly, the exposed dura should be completely intact and still fully covering the brain. In some cases, the dura may be partially removed with the skull during the craniectomy, which may indicate that the dura was inadvertently damaged during the craniectomy, or that the dura is extremely adherent to the skull. Using Adson forceps, we grab a section of the dura, lift it away from the brain, and snip into it using curved blunt-tip dissection scissors (**Figure 2**). We then use this opening to cut away the dura on both hemispheres of the brain (**Figure 3**). The falx cerebri is also removed, cutting at the anterior attachment and gently pulling to further the separation using the natural perforations between the hemispheres. Removal of the initial anterior most part of the falx cerebri often requires slight retraction of the frontal lobes. The same is done to the dura surrounding the cerebellum, ensuring the dura is pulled away from the cerebellum before the initial incision is made in both hemispheres to prevent damage. The removal of the tentorium cerebelli is one of the most challenging aspects and it is critical to fully separate it laterally prior to final brain extraction, to avoid damage to the posterior aspects of the cerebellum during the subsequent final extraction.

### Nerve removal, spinal cord transection and final extraction

**Figure 4:**
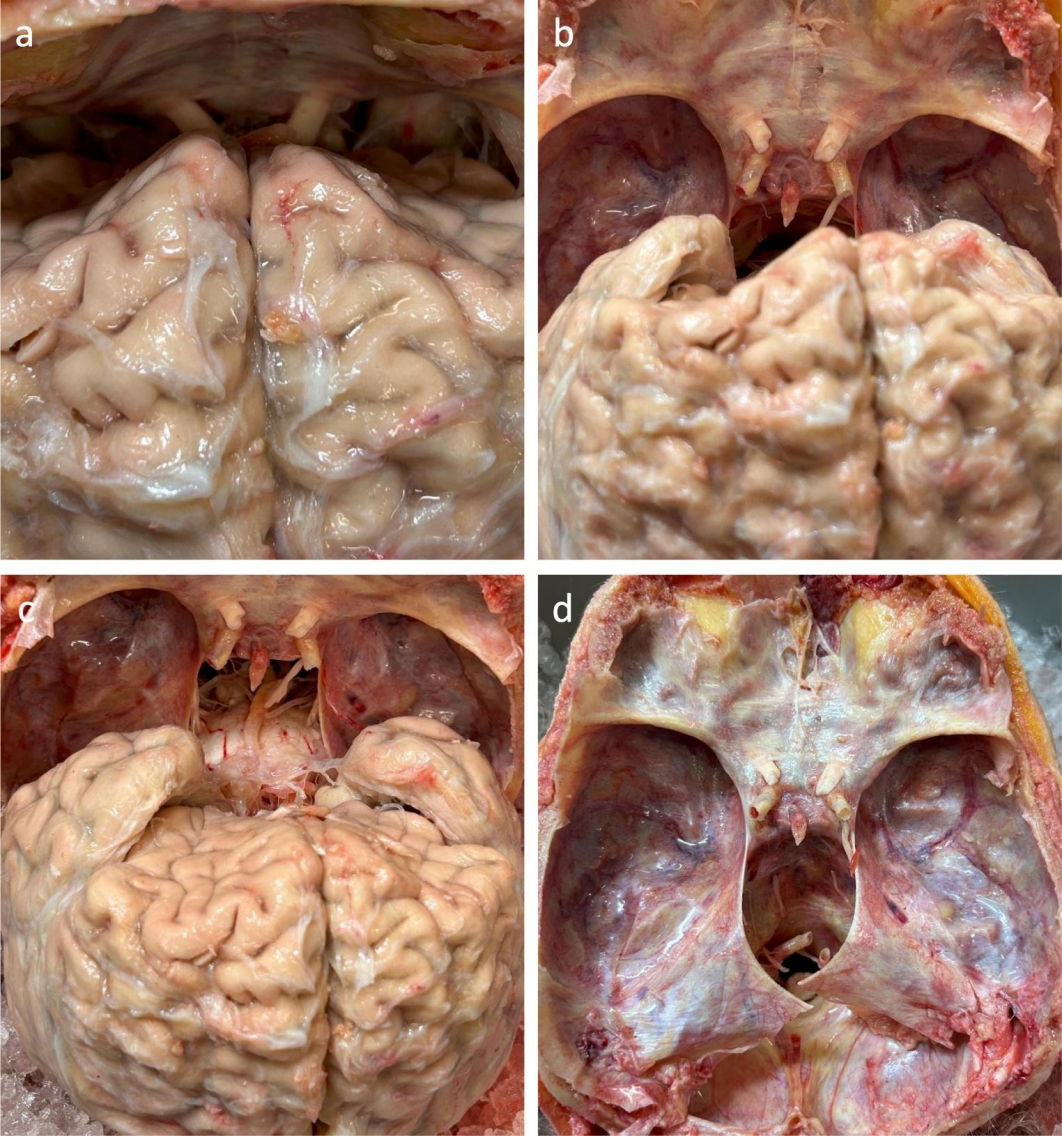
Sequential images during the nerve removal and final brain extraction procedure in one example case. **a**: The cephalon is positioned prone so the frontal lobes fall away from the frontal bone. **b**: The stumps of the optic nerves and internal carotid arteries are visible after they have been separated from the brain. **c**: The brain after falling further posteriorly out of the cranium. The brainstem and nearby structures are more visible. **d**: View into the empty cranial vault after the brain removal, showing the residual attachments of the dura, nerves, and blood vessels that were separated. Donor #297, a 90+-year-old female donor with a PMI of 72 hours.

Once the dura is removed, the cephalon can be repositioned to lie prone, allowing the brain to fall away from the interior of the frontal bone. We then manually retract the brain further away from the interior of the frontal bone to expose the optic nerves (**Figure 4**). The optic nerves are cut using blunt-tip dissection scissors. The remaining cranial nerve attachments are detached via digital manipulation. Detachment of these nerves should give tactile feedback in the form of a "popping" sensation.

Once the cranial nerves are detached, we then begin the last step of the extraction process, which is detaching the spinal cord from the foramen magnum and fully excising the brain from the cranial fossa. This is accomplished by keeping the brain retracted after detaching the cranial nerves and inserting long dissection scissors into the spinal canal, snipping the cord as far away as possible from the base of the brain, to retain as much of the spinal cord connected as feasible. This cut frees what should be the last attachment of the brain to the cranium. Further posteriorly angling the cephalon will allow the brain to gently fall backwards out of the cranium. If the dura surrounding the cerebellum was not fully removed, there may be some dura still holding the cerebellum in the skull. This may be snipped with long dissection scissors to prevent damage to the cerebellum. When working with the whole body intact, the head is commonly tilted back over a mortuary head block and the brain is eased out of the cranial vault after the nerves and spinal cord are cut.

### Brain immersion fixation

**Figure 5:**
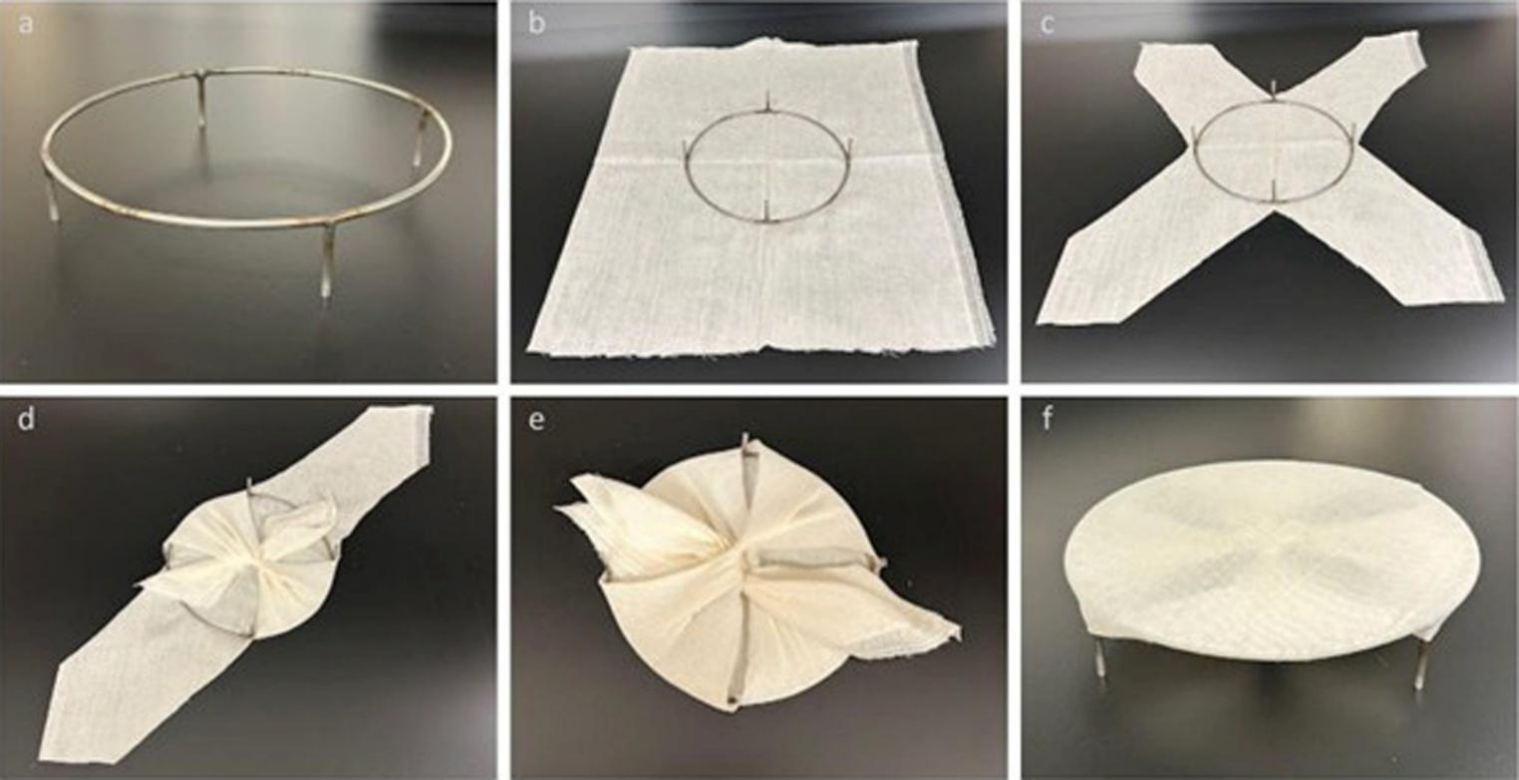
Sequential steps in applying the cheesecloth to the suspension apparatus. **a**: A stainless-steel stand with 1.5" support legs. **b**: The frame is placed upside down on top of two layers of cloth. **c**: The cloth is cut into an X-pattern. **d**: The opposite edges of the cloth are folded inward and tied at the center. **e**: The remaining pieces are tied at the center, followed by double knotting to hold tension. **f**: The assembly is finalized with the cheesecloth stretched across the apparatus.

Once the brain is fully isolated, it is placed in a 7.5-liter high-density polyethylene (HDPE) container with approximately 4 liters of neutral buffered formalin (either 10 % or 20 %) so that it is fully immersed for further fixation. A suspension apparatus is placed within this container to keep the brain suspended in neutral buffered formalin. This prevents the bottom of the brain from resting against the bottom of the container, which would impede fixative penetration and could distort the shape of the tissue. To create this apparatus, a simple frame was crafted from 3/16" stainless steel rod and bent into a ring approximately 20 cm in diameter with four welded legs standing about 1.5" tall (**Figure 5**, **Table 2**). This size allows the apparatus to fit at the bottom of the container while still allowing easy placement and removal. The frame is covered with two layers of cheesecloth cut in an X pattern, with the flaps tied together underneath to create a flexible yet sturdy surface. This arrangement allows the brain to rest securely in place while absorbing fixative solution from all sides, as the cheesecloth does not meaningfully impede penetration. It also allows a magnetic stir bar to be placed beneath the frame, which is used to gently circulate the fixative solution.

**Table 1:** Supplies and reagents used for immersion fixation of the brain after removal, using the primary suspension apparatus.

Equipment	Supplier	Product identifier
2 Gallon HDPE Container	Consolidated Plastics	B0BBP76WVJ
Magnetic Stir Table (50L) with Magnetic Stir Bar	Joan Lab Equipment Co.	B0DK571DJ4
3/16" Stainless steel rod	Industrial Metal Supply	18R0188
Cheese cloth	Cotton Farm From Med	B08HRZ6V88
10 % Neutral buffered formalin	Azer Scientific	NBF55G
20 % Neutral buffered formalin	Fisher Scientific	STL286205

**Figure 6:**
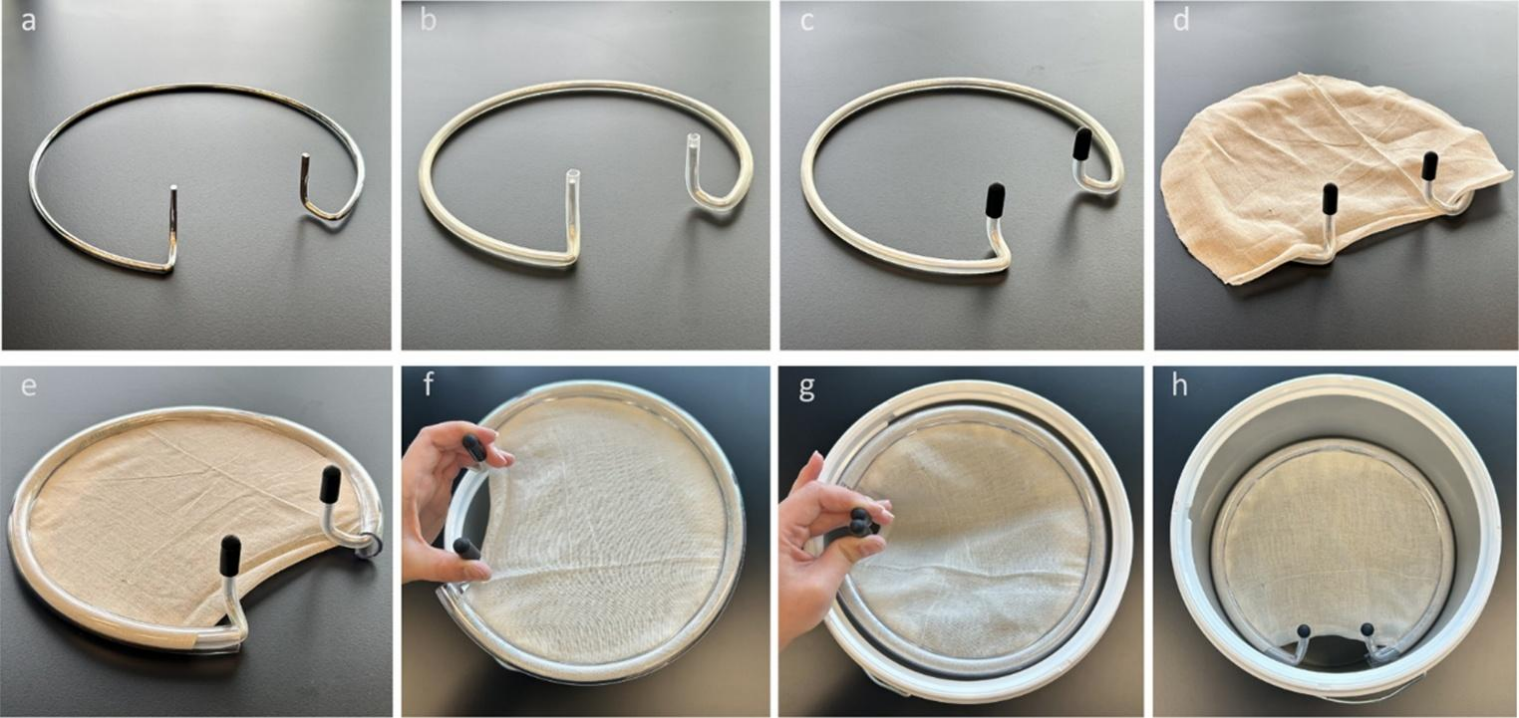
Sequential steps in creating the suspension apparatus design #2. **a**: A steel expansion ring was taken from an embroidery set and the handles were bent in an upward direction with pliers. **b**: The ring is covered with 7x10 mm silicone tubing. **c**: The ends are closed with silicone end caps. **d**: Cheesecloth is laid over the top. **e**: The 3/8" x 3/32" tubing is slit on one side and placed around the ring to tightly enclose the cheesecloth. **f**: The assembly is finalized and ready to be placed in the bucket. **g**: To insert, the handles are squeezed together and the apparatus is set at the desired depth. **h**: Releasing the handles allows the ring to expand slightly, holding it in place by its expansion force and the friction of the outer tubing against the inner wall of the bucket.

We also created a different apparatus design to hold the brain above the bottom of the container during immersion, using more easily sourced items (**Figure 6**, **Table 3**).

**Table 3:** Supplies for immersion fixation of the brain after removal with the suspension apparatus design #2.

Equipment	Supplier	Product identifier
7 mm x 10 mm Pure Silicone Rubber Tubing	Hooshing	B08PTWPXNW
3/8" Peroxided-Cured Silicone Tubing	Cole-Parmer	B0FPHPT5TY
3/16" Stainless steel rod	Industrial Metal Supply	18R0188
8.8" x 8.6" x 0.9" Embroidery Hoop Expansion Ring	Olycraft	TOOL-WH0001-35B-US13
5/16" Silicone End Caps	CQRobot	CQRGJHM800B

### Histology

We collected a sample from the frontal lobe of a 79-year-old male donor with a PMI of 23 hours that had a craniectomy artifact in this area caused by the oscillating saw during the craniectomy. Brain tissue sampled for light microscopy was placed into a cassette for processing and embedded in paraffin. A paraffin-embedded brain section 6 μm thick was baked, deparaffinized, and stained for Hematoxylin and Eosin (H&E). A digital image of the stained section was captured at 40X as whole slide images (WSIs) using the Aperio GT450 high-resolution scanner (Leica Biosystems).

### Test of diffusion speed with in situ fixation

To assess whether* in situ* immersion fixation affects the diffusion rate of chemicals into brain tissue, we compared two non-perfused donor brains: one immersion fixed *in situ* (i.e. the brain remaining in the cranial vault after removal of the calvarium and dura on the surface of the brain) and one immersion fixed *ex situ* (i.e. with the brain fully extracted and suspended in fixative). Both brains were immersed in 10 % neutral buffered formalin containing iohexol 300 mgI/mL (Omnipaque, Med-Plus 0407-1413-63) as a CT-visible tracer to visualize the relative penetration rate. The *in situ* brain was immersed in approximately 5 L of formalin with iohexol at ~3 mgI/mL, while the *ex situ* brain was placed at the bottom of a container immersed in approximately 4 L with iohexol at ~3.75 mgI/mL (without the suspension apparatus). Magnetic stirring of the fluid was not used in either case. Serial CT scans were acquired daily for several days using a CT scanner (OmniTom® Elite; Neurologica, Danvers, MA). Images were reviewed using the Osimis Web Viewer. Diffusion was assessed qualitatively by visual inspection of contrast penetration into the brain parenchyma across timepoints.

### Data analysis

For each case, we measured the time to perform each step and two types of artifactual tissue disruption. First, craniectomy artifacts were defined as tissue disruption caused by contact with the oscillating saw blade or osteotome during the craniectomy. Second, skull edge artifacts were defined as tissue disruption caused by contact of the brain with the cut edges of the skull during brain removal.

## Results

### Development of our brain extraction method

We made changes to our brain extraction protocol over time through iterative refinement. We initially attempted to perform a standard circumferential craniectomy without a midline cut, but we found that this does not allow for sufficient access to the dura, especially the falx cerebri and falx cerebelli, and therefore increases the risk of mechanical damage to the brain tissue during removal of the skull. Therefore, we added a sagittal cut through the midline of the skull, which allows for a better ability to remove the dura, while minimizing the risk of craniectomy artifact, because it is made in the natural space created by the hemispheres. In our experience, adding the midline cut may actually reduce the total procedural time, because it decreases the amount of time needed to do the dural removal, potentially more than making up for the amount of time needed to make the midline skull cut itself. However, we do not have the data to quantify this hypothesis.

Another procedural modification we adopted is to use the oscillating saw to create a shallow guiding groove, rather than cutting completely through the bone. We then complete the separation with an osteotome and mallet. We found that this provides better control and that it qualitatively appears to decrease the risk of brain craniectomy artifact.

Finally, we position our circumferential cut approximately 3–4 cm below the occipital protuberance, which is more inferior than in some previously described protocols (23). Notably, we do not perform a separate wedge cut (or "hinge cut") in the occipital region, which we found creates more sharp edges of the skull that could potentially damage the brain or pose an injury risk to the staff member performing the procedure. The more inferior occipital cut that we perform exposes the posterior fossa and cerebellum to view, providing better access to the tentorium cerebelli. If the cut were to instead be made more superiorly, then the cerebellum would remain enclosed within the skull and would only be able to be accessed from an anterior approach after elevating the cerebrum. From our observations, attempting to cut the tentorium cerebelli from this anterior position, with the cerebellum still seated in the posterior fossa, increases the risk of inadvertent dissection artifact in the cerebellum due to limited visualization and constrained instrument angles. By first removing the dura from the posterior aspect with clear visualization, we can then reposition the cephalon prone, allowing the brain to fall away from the frontal bone due to gravity. This also reduces the force required to elevate the frontal lobes and provides a more controlled brain extraction.

### Speed of brain extraction

**Figure 7:**
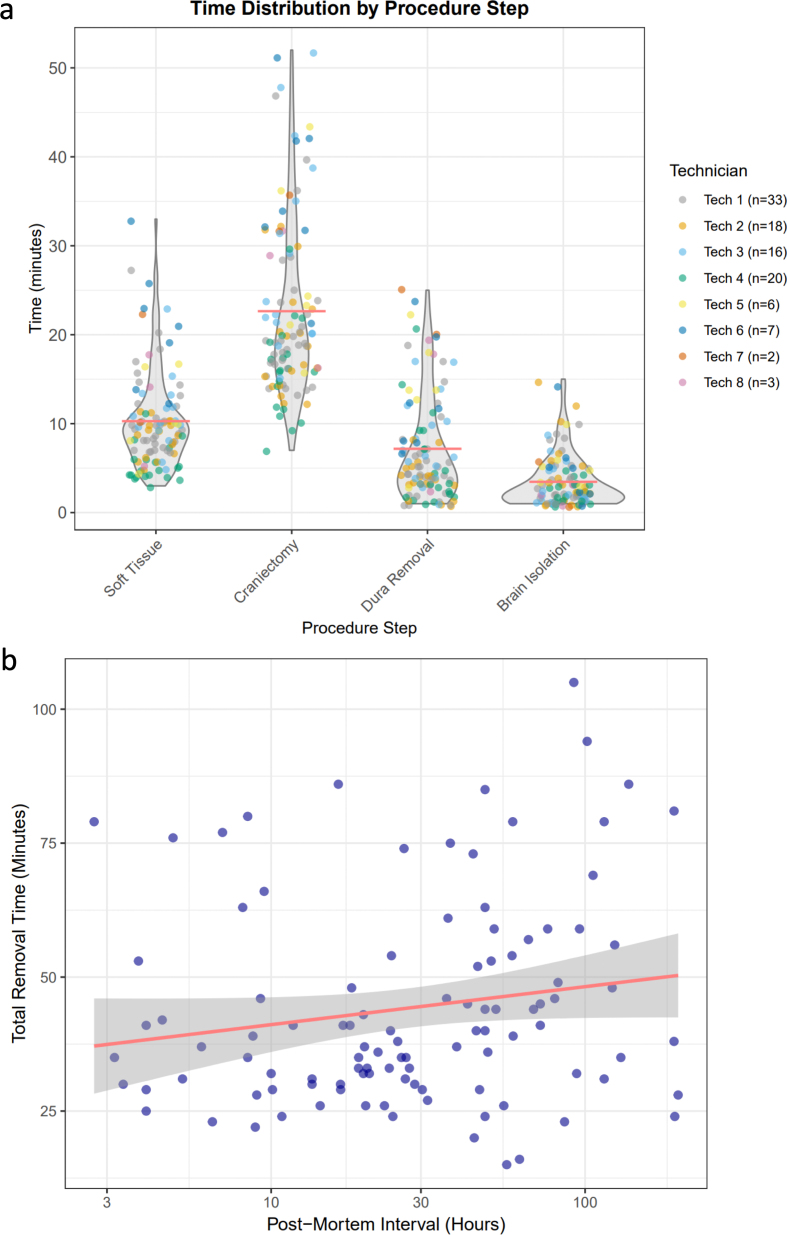
**a**: Time distribution by procedure step during brain extraction. Violin plots showing the distribution of time (in minutes) required for each major step of the brain extraction procedure: soft tissue removal, craniectomy, dura removal, and brain isolation. Each dot represents an individual case, color-coded by the technician performing the procedure. For visualization purposes, one outlier case with a recorded craniectomy time of 95 minutes was removed. Red horizontal lines indicate group means. Data points are horizontally jittered for visibility. **b**: Scatter plot showing the relationship between the PMI and the total brain extraction time. Each point represents an individual case. The x-axis is displayed on a logarithmic scale to accommodate the wide range of PMI values (2–100+ hours). The red line shows a linear regression fit with 95 % confidence intervals (grey shaded region). These plots were made using ggplot2.

We recorded the time it takes to perform each different step of the procedure (**Figure 7a**). We found that the craniectomy step required the most time, with a mean of 23.3 minutes (SD = 12.0, range: 7–95 minutes). Soft tissue removal averaged 10.3 minutes (SD = 5.5). Dura removal and brain isolation were the fastest steps, averaging 7.2 minutes (SD = 6.0) and 3.5 minutes (SD = 2.9), respectively. The total procedure time across all steps was on average 44.2 minutes (SD = 19.2, range: 15–105 minutes). Although there was within-technician variability, some technicians tended to perform some steps generally faster than other technicians, likely in part due to differences in prior experience (**Figure 7a**). We also tested the relationship between the PMI prior to the procedure and the time it took to extract the brain, finding that there was no significant rank correlation between the PMI and the extraction time (ρ = 0.19, p = 0.052; **Figure 7b**).

### Artifactual tissue disruption during extraction

Craniectomy artifacts, primarily caused by the oscillating saw, occurred in 65 of the 105 cases (61.9 %), with a mean of 0.98 craniectomy artifacts per case (SD = 0.99, range: 0–4). We note that craniectomy artifacts also differ in transverse length and depth in the tissue, which we did not quantify here. Among technicians with at least 5 cases, craniectomy artifact frequency did not differ significantly across technicians in our sample (Kruskal-Wallis H = 7.49, p = 0.19). In contrast, the total procedure time did vary significantly by technician (Kruskal-Wallis H = 42.6, p < 0.001). Notably, the fastest technicians in our sample were not the ones with the most craniectomy artifacts. For example, one technician (Tech 4) who had 20 cases in this series, but by far the most prior experience, had both the shortest mean procedure time (29.1 minutes) and slightly below-average craniectomy artifacts (0.90 per case).

Skull edge artifacts were more difficult for us to code reliably. Recorders appeared to interpret these tissue distortions differently, as they are often subtle and harder to visualize than frank craniectomy artifacts. Also, the degree to which they cause histological damage to underlying tissue can be unclear. Nevertheless, we recorded them and we therefore report this data for completeness. We observed a mean of 1.04 skull edge artifacts per case (SD = 0.87, range: 0–4), with 78.1 % of cases having at least one apparent skull edge artifact.

**Figure 8: Example histological effects of craniectomy artifact:**
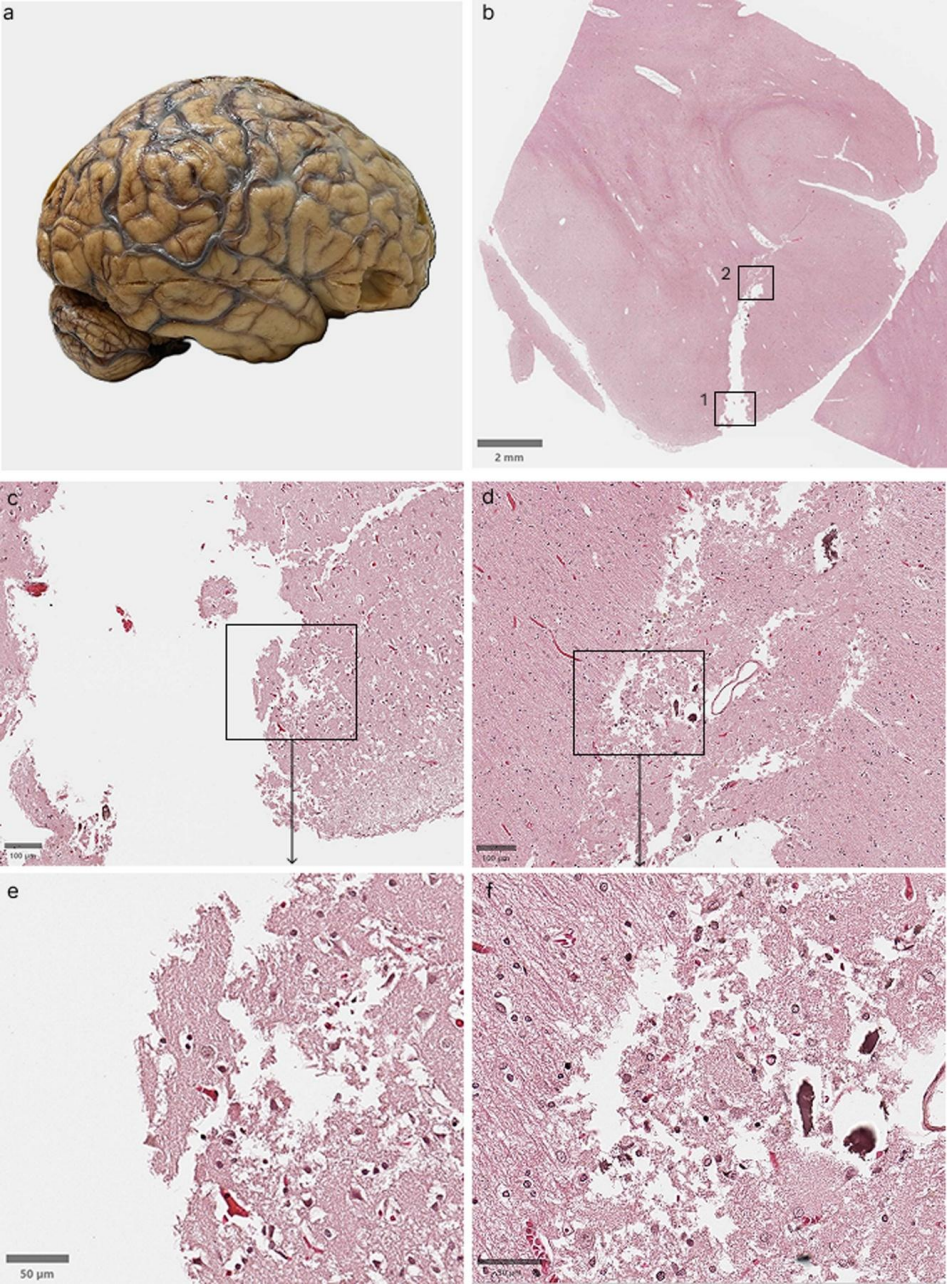
Tissue from a 79-year-old male donor with a PMI of 23 hours (donor ID #290). The brain was perfusion fixed prior to extraction. **a:** Gross photograph of the brain showing the site of the transverse craniectomy artifact on the right hemisphere, and the square area where the biopsy was taken from. **b:** H&E-stained whole slide image of a section through the craniectomy artifact site, with boxes indicating regions shown in panels **c**–**f** (box #1: **c** and **e**, box #2: **d** and **f**). Scale bar: 2 mm. **c, d:** Intermediate magnification views showing disrupted craniectomy artifact margins. Scale bars: 100 μm. **e, f:** Higher magnification views showing the degree of tissue disruption at the cellular level. Scale bars: 50 μm.

We next examined the histological effects of a craniectomy artifact in one case using H&E staining (**Figure 8**). At lower magnification, an area of tissue loss and/or displacement corresponding to the site of the craniectomy artifact can be seen in the cortical surface, measuring approximately 4.5 mm in depth. At higher magnification, the tissue at the craniectomy artifact margins appears jagged and disrupted, with displacement of parenchymal fragments. Deeper to the base of the cut, the zone of apparent tissue disruption is present beyond the craniectomy artifact site, possibly as a result of thermal and mechanical damage caused by the oscillating saw to the adjacent parenchyma.

### Technician training procedure

**Figure 9:**
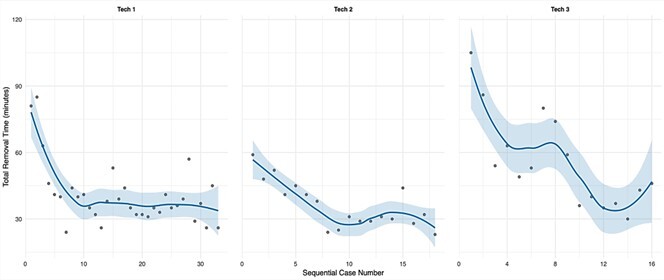
Learning curves for brain extraction procedure by technician. Total removal time (minutes) plotted against sequential case numbers for the technicians with at least 10 cases and without significant prior experience before this case series (n = 3 technicians). Each point represents an individual case. Blue lines show locally estimated scatterplot smoothing regression fits, while shaded regions indicate 95 % confidence intervals. This plot was made using ggplot2.

We found that the total removal time decreased with technician experience, with the steepest improvement occurring within the first 5–10 cases (**Figure 9**). After approximately 5–10 cases, removal times generally stabilized around 30–40 minutes. The decrease in the time required to perform an extraction over time was correlated with a subjective feeling of greater anatomical familiarity and confidence in technique among the technicians.

### Immersion diffusion speed with in situ fixation

**Figure 10: Comparison of fixative diffusion:**
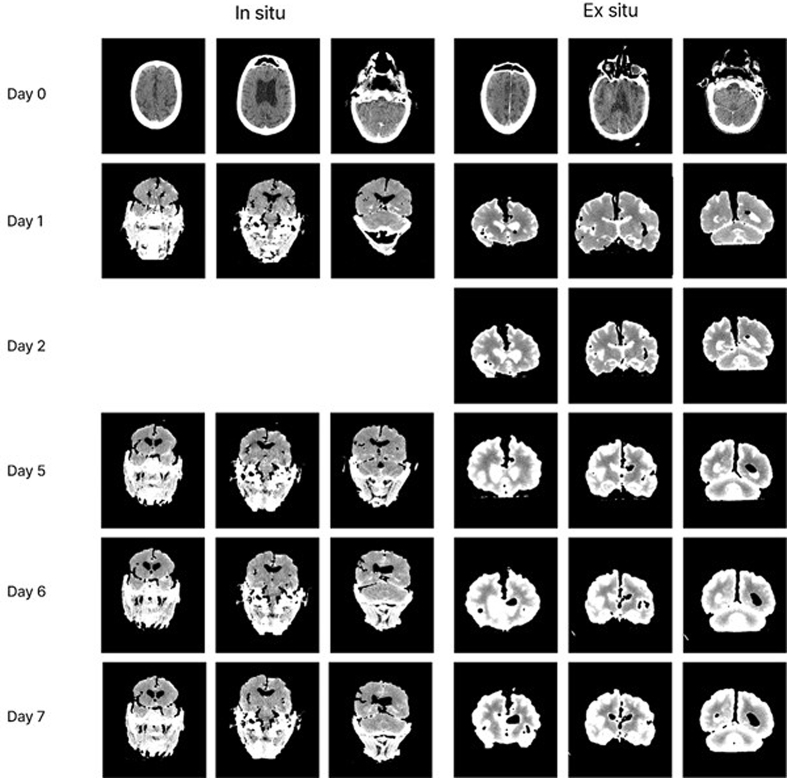
Serial CT images of two non-perfused human brains immersed in iohexol-containing 10 % neutral buffered formalin, scanned daily over several days. Left three columns: brain fixed *in situ* (calvarium and surface dura removed, brain remaining in cranial vault; donor ID #287, brain from a 62-year-old male with a PMI of 25 hours). Right three columns: brain fixed *ex situ* (fully extracted and suspended in fixative; donor ID #285, brain from an 89-year-old male with a PMI of 80 hours). Each row represents a different day of immersion. Bright signal on the CT scan indicates iohexol penetration.

In two cases, we compared the diffusion rates of a contrast agent (iohexol) during *in situ* versus *ex situ* immersion fixation (**Figure 10**). The brain immersed *ex situ* showed a rim of bright signal at the cortical surface by Day 1 that progressively expanded inward, with contrast penetrating several centimeters by Day 5. In contrast, the brain immersed *in situ* showed minimal parenchymal uptake throughout the observation period. While iohexol (~821 Da) is larger than formaldehyde (~30 Da), this finding suggests that *in situ* fixation likely slows fixative diffusion as well, presumably due to a smaller surface area allowing for diffusion when the brain is still inside of the cranial vault. Note that the *ex situ* brain was resting at the bottom of the container rather than on a suspension apparatus, so some inferior surface area was also occluded, yet diffusion was still markedly faster than in the* in situ* condition. However, because we only have one observation from each group, this comparison should be regarded as preliminary.

### Variations in anatomy

**Figure 11:**
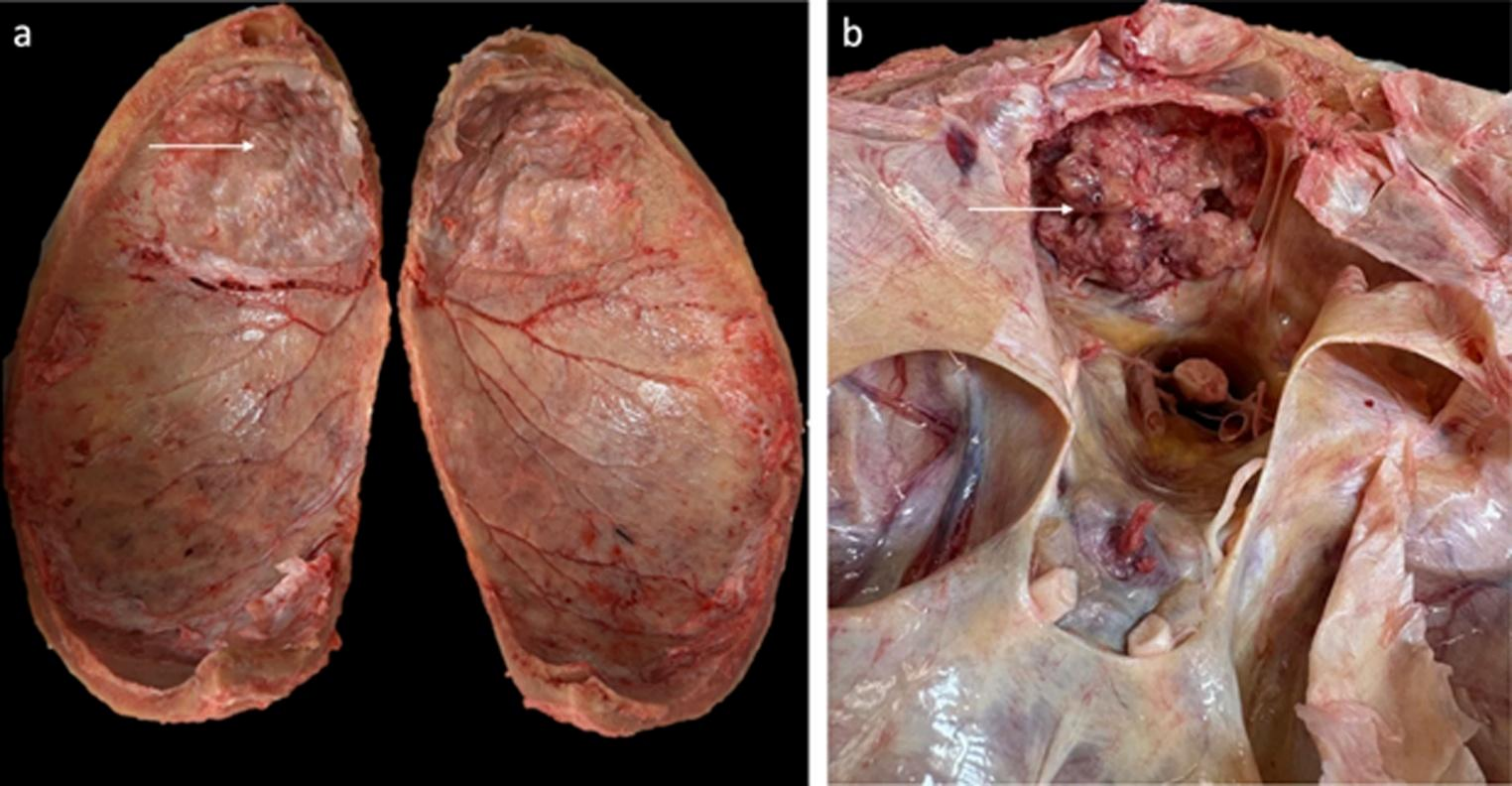
Example anatomical variations encountered during brain extraction. **a**: Interior view of the removed calvarium showing hyperostosis frontalis interna, a benign overgrowth of the inner table of the frontal bone (white arrow). **b**: View into the cranial cavity showing residual brain tumor tissue (white arrow) adherent to surgical mesh from prior craniotomy. Complete removal of the affected tissue was not possible without excision of the surrounding skull.

Anatomical variations encountered during brain extraction can affect the procedure. In our experience, some anatomical variations have minimal impact on the extraction procedure, while others present significant challenges. One important variable is the degree to which the dura is attached to the skull. We have found that in younger donors, this is minimal, while it becomes more adherent in older donors, corroborating previous reports (29,30). Another example of anatomical variation is hyperostosis frontalis interna, which is a benign overgrowth of the inner table of the frontal bone (31). This condition was observed in one donor (**Figure 11a**). This did not substantially impede brain extraction or appear to increase the risk of tissue artifacts. In contrast, prior neurosurgery can pose more significant obstacles. In one case, a donor with a history of brain tumor resection had residual tumor tissue adherent to a surgical mesh embedded in the skull (**Figure 11b**). This attachment prevented complete removal of the affected brain region using our standard dissection techniques. The full extraction of this tissue would have required *en bloc* excision of the overlying skull segment.

## Discussion

In this study, we describe our approach to brain extraction in the context of whole-body donation. Three procedural modifications distinguish our approach from some other protocols. First, we incorporate a midline sagittal cut through the skull in addition to the standard circumferential cut. While this adds an additional step, we qualitatively found that it can reduce the potential for damage during extraction, and that it can save procedural time by making subsequent dura removal easier. Another recent study also reported benefits of a mid-sagittal cut (9). Second, we use the oscillating saw to create only a guiding groove rather than cutting completely through the bone, prior to completing the separation with an osteotome and mallet. In our experience, this appears to provide better tactile control and reduce the risk of inadvertent craniectomy artifacts. Third, we position the posterior aspect of our circumferential cut approximately 3–4 cm below the occipital protuberance, which is more inferior than in some other protocols. This exposes the posterior fossa and allows dura removal to be performed from the posterior aspect, allowing for better visualization, which we believe can help to reduce dissection artifacts. This posterior approach to cutting the tentorium cerebelli has been previously reported as well (6,7), but instead of creating a separate "occipital wedge" in the skull, we favor simply making the circumferential incision more inferior instead.

Our timing data shows that there is a moderate degree of case-to-case variation in how long the procedure takes, especially in the craniectomy step. This variability is due to many factors, including differences in skull thickness, skull or brain pathology, dural adhesion, and technician experience. Our learning curve analysis suggests that with regular practice, in our sample, technicians can achieve procedural fluency after approximately 5–10 cases, after which removal times tended to take around 30-40 minutes. Qualitatively, it is obvious that there could be a theoretical trade-off between the duration of the procedure and the risk of tissue artifacts in the brain, because there are different steps in the procedure wherein one could take more or less time. However, we did not see this as a major factor in our data, perhaps because our technicians naturally prioritized tissue integrity over speed, or because of our limited sample size. We note that the development of virtual reality training tools has the potential to accelerate the learning curve for brain extraction (32). In the more distant future, it may also be possible to automate aspects of the brain extraction process with robotics, but it is currently expensive and challenging to automate even partial craniectomy of rodent skulls (33), which is a much easier problem.

We also performed preliminary testing of an *in situ* immersion fixation protocol as a potential method to help stiffen relatively softer brains prior to extraction, reasoning that some degree of surface fixation might help to mitigate mechanical damage during removal. Low stiffness is very commonly seen in brains with longer PMIs, especially those that have not been refrigerated, because refrigeration both slows decomposition and helps stiffen the brain. However, with CT imaging, we found that this approach substantially slows the diffusion of chemicals into the brain parenchyma, thus delaying the fixation of deeper structures. If these preliminary results are corroborated, they suggest that this approach likely only makes sense if the *in situ* immersion period is kept brief, or if perfusion fixation quality is very high (in which case fixation via immersion is not as necessary). This approach is also only feasible in the context of whole-body donation and whole brain fixation (as opposed to hemi-secting the brain and cryopreserving half of it), which are both major additional practical barriers.

Our study has several limitations. First, our protocol requires whole body donation rather than brain-only donation, which limits its applicability to most existing brain banking operations. The ability to isolate the cephalon enables positioning advantages that may not translate to standard brain extraction performed with the body intact. However, several elements of our approach could likely also be applied in more conventional brain extraction settings. Second, we did not systematically compare our method to alternative protocols in a controlled manner. Instead, our conclusions about which approaches lead to better outcomes are based on our subjective experience, which is less reliable than randomized studies. Related to that, because we iteratively refined our technique over time and did not systematically record how these changes were made over time, we could not quantitatively assess whether a given change led to improvements in outcomes. Third, our assessment of tissue artifacts relied on coarse-grained, qualitative macroscopic observation and very limited histological sampling of one sample with a craniectomy artifact. Future studies could more thoroughly investigate the degree of histological artifacts that may be differentially present based on the method and effectiveness of brain extraction, such as dark neurons (14).

## Conclusions

Brain extraction is a fundamental aspect of brain banking. Qualitatively, our experience suggests that certain procedural refinements, such as using a midline sagittal cut, separating the skull with an osteotome, and cutting the tentorium cerebelli from a posterior approach, can improve the effectiveness of brain extraction. Based on our timing data, technicians appear to reach a basic proficiency after approximately 5–10 cases. Future directions include extending our methods to banking additional structures such as the spinal cord, as well as developing better tools for more rapid and artifact-free brain removal. We anticipate that improving brain extraction methods may support downstream research applications ranging from molecular profiling to connectomics, ultimately advancing our understanding of neurological and psychiatric disease.

## Author contributions

J.F.C., K.F., and A.T.M. conceptualized the study. M.W., A.B., L.P., S.D., A.P., G.A.T., M.G., E.T., C.D.S., and A.T.M. performed brain extraction and histology experiments. M.W. and A.T.M. performed data analysis. M.W., A.B., and A.T.M. wrote the initial draft of the manuscript. All authors reviewed the manuscript and approved the final manuscript.

## Declaration of generative AI technologies

In the preparation of this manuscript, the authors used Claude (Anthropic) for both programming assistance and to improve the manuscript’s language. All AI tool-assisted content was reviewed and edited by the authors, who take full responsibility for the final publication.

## Data availability

Whole slide image data can be accessed in a public repository on Zenodo, available here: https://zenodo.org/communities/brainextraction. Code and data is available at https://github.com/andymckenzie/Brain_extraction.

## Conflict of interest statement

Mads Wolf, Autumn Beck, Laura Paredes, Sarah Darcy, Alexander Parra, Gabriel Taylor, Macy Garrood, and Andrew McKenzie are employees of Sparks Brain Preservation, a non-profit brain preservation organization.

## References

[R1] McKee AC. Brain banking: basic science methods. Alzheimer Dis Assoc Disord. 1999;13 Suppl 1: S39-44. PMID:10369517 10369517

[R2] Haroutunian V, Davis KL. Issues and perspectives on brain tissue banking. Curr Psychiatry Rep. 2002 Aug 1;4(4):233–4. 10.1007/s11920-996-0039-6 12126590

[R3] Beach TG, Sue LI, Walker DG, Roher AE, Lue L, Vedders L, et al. The Sun Health Research Institute Brain Donation Program: Description and Experience, 1987–2007. Cell Tissue Bank. 2008;9(3):229–45. 10.1007/s10561-008-9067-2 PMC2493521 18347928

[R4] Danner B, Gonzalez AD, Corbett WC, Alhneif M, Etemadmoghadam S, Parker-Garza J, et al. Brain banking in the United States and Europe: Importance, challenges, and future trends. J Neuropathol Exp Neurol. 2024 Mar 20;83(4):219–29. 10.1093/jnen/nlae014 PMC10951968 38506125

[R5] Jumlongkul A, Chutivongse P. Robotic-Assisted Surgery for Cadaveric Skull Opening: A New Method of Autopsy Procedure. Front Robot AI. 2020;7:622083. 10.3389/frobt.2020.622083 PMC7925889 33681298

[R6] Felle P, Lockman AK, Kellaghan P. Removal of the brain for teaching and examination. Clin Anat. 1995;8(5):363–5. 10.1002/ca.980080510 8535970

[R7] Long J, Roberts DJH, Pickering JD. Preservation of cranial nerves during removal of the brain for an enhanced student experience in neuroanatomy classes. Clin Anat. 2014 Jan;27(1):20–4. 10.1002/ca.22356 24318012

[R8] Hlavac RJ, Klaus R, Betts K, Smith SM, Stabio ME. Novel dissection of the central nervous system to bridge gross anatomy and neuroscience for an integrated medical curriculum. Anat Sci Educ. 2018 Mar;11(2):185–95. 10.1002/ase.1721 28817239

[R9] Robertson EM, Allison SM, Mueller CM, Ferriby AC, Roth AR, Batra R. Exploring effectiveness in brain removal techniques: A comparison of approaches. Anat Sci Educ. 2024;17(1):147–56. 10.1002/ase.2333 37638528

[R10] Loomis M, Nikirk J, Loomis T, Wooten D, Prada Iii G. Brain Removal With an Occipital Hinge Preserving Overlying Anatomy. Cureus. 2024 Oct;16(10 ):e72377. 10.7759/cureus.72377 PMC11586063 39583428

[R11] Żytkowski A, Dębski J, Orkisz S. Techniques of skull opening and brain extraction: Contemporary approaches and technical considerations. Translational Research in Anatomy. 2025 Jun 1;39:100401. 10.1016/j.tria.2025.100401

[R12] Krassner MM, Kauffman J, Sowa A, Cialowicz K, Walsh S, Farrell K, et al. Postmortem changes in brain cell structure: a review. Free Neuropathol. 2023 Jan;4:4–10. 10.17879/freeneuropathology-2023-4790 PMC10294569 37384330

[R13] Hooper JE. Rapid Autopsy Programs and Research Support: The Pre- and Post-COVID-19 Environments. AJSP Rev Rep. 2021;26(2):100–7. PMID:33718610 33718610 PMC7954201

[R14] Cammermeyer J. Is the solitary dark neuron a manifestation of postmortem trauma to the brain inadequately fixed by perfusion? Histochemistry. 1978 Jun 9;56(2):97–115. 10.1007/BF00508437 97249

[R15] Weickenmeier J, de Rooij R, Budday S, Steinmann P, Ovaert TC, Kuhl E. Brain stiffness increases with myelin content. Acta Biomater. 2016 Sep 15;42:265–72. 10.1016/j.actbio.2016.07.040 27475531

[R16] Hayman J, Oxenham M. Estimation of the time since death in decomposed bodies found in Australian conditions. Australian Journal of Forensic Sciences. 2017 Jan 2;49(1):31–44. 10.1080/00450618.2015.1128972

[R17] Garrood M, Keberle A, Taylor GA, Thorn EL, Sanctis CD, Farrell K, et al. Mechanical perfusion in brain banking: methods of assessment and relationship to the postmortem interval. Free Neuropathol. 2025;6:20. 10.17879/freeneuropathology-2025-8880 PMC12557960 41159117

[R18] Rengachary SS, Colen C, Dass K, Guthikonda M. Development of anatomic science in the late middle ages: the roles played by Mondino de Liuzzi and Guido da Vigevano. Neurosurgery. 2009 Oct;65(4):787–93; discussion 793-794. 10.1227/01.NEU.0000324991.45949.E4 19834385

[R19] Giménez-Roldán S. Andreas Vesalius and the brain: limitations of De humani corporis fabrica libri septem and some comments on the matter. Neurosci Hist. 2020;8:76–86.

[R20] Sanan A, Abdel Aziz KM, Janjua RM, van Loveren HR, Keller JT. Colored silicone injection for use in neurosurgical dissections: anatomic technical note. Neurosurgery. 1999 Nov;45(5):1267–71; discussion 1271-1274. 10.1097/00006123-199911000-00058 10549950

[R21] McFadden WC, Walsh H, Richter F, Soudant C, Bryce CH, Hof PR, et al. Perfusion fixation in brain banking: a systematic review. Acta Neuropathol Commun. 2019 Sep 5;7(1):146. 10.1186/s40478-019-0799-y PMC6728946 31488214

[R22] Mignucci-Jiménez G, Xu Y, On TJ, Abramov I, Houlihan LM, Rahmani R, et al. Toward an optimal cadaveric brain model for neurosurgical education: assessment of preservation, parenchyma, vascular injection, and imaging. Neurosurg Rev. 2024 Apr 25;47(1):190. 10.1007/s10143-024-02363-7 38658446

[R23] Adams JH, Murray MF. Atlas of post-mortem techniques in neuropathology. Cambridge University Press; 1982. 10.1017/CBO9780511735479

[R24] Knudsen SK, Mørk S, Øen EO. A novel method for in situ fixation of whale brains. J Neurosci Methods. 2002 Oct 15;120(1):35–44. 02)00182-6" target="_blank"> 02)00182-6 10.1016/s0165-0270(12351205

[R25] Avelino-De-Souza K, Valdevino G de CM, Melo-Santos G, Mota B, Da Silva VMF. How to remove the brain of Amazonian manatee (Trichechus inunguis) calves preserving the skull for morphological analysis. Acta Amaz. 2025 Apr 7;55:e55bc23384. 10.1590/1809-4392202303841

[R26] Snyder JM, Radaelli E, Goeken A, Businga T, Boyden AW, Karandikar NJ, et al. Perfusion with 10 % neutral-buffered formalin is equivalent to 4 % paraformaldehyde for histopathology and immunohistochemistry in a mouse model of experimental autoimmune encephalomyelitis. Veterinary pathology. 2022 Feb 7;59( 3):498. 10.1177/03009858221075588 PMC9364762 35130806

[R27] Musigazi GU, De Vleeschauwer S, Sciot R, Verbeken E, Depreitere B. Brain perfusion fixation in male pigs using a safer closed system. Lab Anim. 2018 Aug 1;52(4):413–7. 10.1177/0023677217752747 29320926

[R28] Insausti R, Insausti AM, Muñoz López M, Medina Lorenzo I, Arroyo-Jiménez MDM, Marcos Rabal MP, et al. Ex vivo, in situ perfusion protocol for human brain fixation compatible with microscopy, MRI techniques, and anatomical studies. Front Neuroanat. 2023;17:1149674. 10.3389/fnana.2023.1149674 PMC10076536 37034833

[R29] Murzin VE, Goriunov VN. [Study of the strength of the adherence of the dura mater to the bones of the skull]. Zh Vopr Neirokhir Im N N Burdenko. 1979;(4):43–7. PMID:484154 484154

[R30] Chen H, Guo Y, Chen SW, Wang G, Cao HL, Chen J, et al. Progressive epidural hematoma in patients with head trauma: incidence, outcome, and risk factors. Emerg Med Int. 2012;2012:134905. 10.1155/2012/134905 PMC3536037 23320175

[R31] Beresheim AC, Hall A. An Investigation of Hyperostosis Frontalis Interna in a Modern Anatomical Body Donor Population. Clin Anat. 2025 Sep 10. 10.1002/ca.70025 PMC12914153 40927897

[R32] Choi I. Autopsy Brain Removal Training Using Virtual Reality Simulation. J Biocommun. 2019 Nov 27;43(2):e16. 10.5210/jbc.v43i2.10225

[R33] Navabi ZS, Peters R, Gulner B, Cherkkil A, Ko E, Dadashi F, et al. Computer vision-guided rapid and precise automated cranial microsurgeries in mice. Sci Adv. 2025 Apr 11;11(15):eadt9693. 10.1126/sciadv.adt9693 PMC11980847 40203110

